# A Qualitative Evaluation of Factors Influencing the Lung Cancer Screening Program Navigator Role

**DOI:** 10.1007/s11606-025-09714-0

**Published:** 2025-07-25

**Authors:** Lucy B. Spalluto, Kemberlee Bonnet, David Schlundt, Carolyn M. Audet, Claudia I. Henschke, David F. Yankelevitz, Sally J. York, Fred Hendler, Robert S. Dittus, Drew Moghanaki, Christianne L. Roumie, Jennifer A. Lewis

**Affiliations:** 1 Geriatric Research Educational and Clinical Center (GRECC) and the VETWISE-LHS Center of Innovation, VA Tennessee Valley Healthcare System, Nashville, TN USA; 2 Department of Radiology and Radiological Sciences, Vanderbilt University Medical Center, Nashville, TN USA; 3 Vanderbilt Ingram Cancer Center, Vanderbilt University Medical Center, Nashville, TN USA; 4 Department of Psychology, Vanderbilt University, Nashville, TN USA; 5 Department of Health Policy, Vanderbilt University Medical Center, Nashville, TN USA; 6 Department of Diagnostic, Molecular and Interventional Radiology, Icahn School of Medicine at Mount Sinai, New York, NY USA; 7 Phoenix Veterans Health Care System, Phoenix, AZ USA; 8 Medicine Service, VA Tennessee Valley Healthcare System, Nashville, TN USA; 9 Department of Medicine, Vanderbilt University Medical Center, Nashville, TN USA; 10 Medicine Service, Robley Rex VA Medical Center, Louisville, KY USA; 11 Radiation Oncology Service, Veterans Affairs Greater Los Angeles Healthcare System, Los Angeles, CA USA; 12 Department of Radiation Oncology, University of California, Los Angeles, CA USA

**Keywords:** lung cancer, screening, low-dose CT, navigator

## Abstract

**Background:**

Lung cancer screening program navigators improve adherence and patient experience. However, little is known about *how* navigators improve program outcomes.

**Objective:**

The aim of this qualitative study was to explore factors influencing the lung cancer screening program navigator role.

**Design:**

From December 2020 to September 2021, we conducted a cross-sectional qualitative study of in-depth interviews in the Veterans Health Administration.

**Participants:**

We interviewed a national sample of healthcare team members involved in lung cancer screening at 10 Veterans Affairs Medical Centers.

**Approach:**

We performed interviews to elicit data on lung cancer screening team and organizational characteristics, barriers to and facilitators of lung cancer screening, and factors influencing the navigator role. We utilized an iterative inductive-deductive approach for qualitative analysis based on the health systems science framework and relational coordination theory.

**Participants:**

We conducted 30 interviews (participation rate = 56%). The 30 participants were predominantly physicians (47%), in primary care (33%), and with 1–10 years of experience in current role (37%).

**Key Results:**

Participants indicated that both structural and relational factors influence the lung cancer screening program navigator role. Specifically, organizational support to strengthen the navigator’s ability to function as a boundary spanner emerged as a key factor that can influence program outcomes such as efficient enrollment practices and program growth.

**Conclusions:**

Healthcare team members involved in lung cancer screening indicated that navigators play an important role in impacting lung cancer screening programs. As lung cancer screening programs continue to evolve and expand, developing organizational structure and relational coordination to support navigators can positively impact program outcomes.

**Supplementary Information:**

The online version contains supplementary material available at 10.1007/s11606-025-09714-0.

## INTRODUCTION

Lung cancer screening with low-dose computed tomography (LDCT) is an effective, evidence-based practice to identify lung cancer in high-risk patients at an earlier, treatable stage.^1,2^ However, lung cancer screening is a complex multi-step process that often relies on navigators to facilitate clinical workflow and coordinate care within an interdisciplinary team of primary care, radiology, and sub-specialty clinicians and staff.^3,4^ A lung cancer screening program navigator facilitates program processes by both providing direct patient care and coordinating program logistics such as tracking a program’s clinical and quality metrics. Program navigators have been shown to improve identification of patients eligible for screening, LDCT utilization, and patient adherence to annual repeat LDCT and can enhance the patient experience.^5–8^ However, little is known about *how* navigators influence lung cancer screening program outcomes.

Program navigators are charged with guiding patients through the health system, helping them overcome barriers to accessing services.^9^ Navigators can also play an important role in improving health outcomes by engaging patients from all backgrounds and education levels, acting as an educator and advocate, and providing support throughout the care cascade.^10^ Recognizing the importance of the navigator role, the Veterans Health Administration’s (VHA) conducted an Enterprise-Wide Initiative (EWI), the Veteran Affairs Partnership to increase Access to Lung cancer Screening (VA-PALS), between 2018 and 2022 that provided funding for 10 Veterans Affairs Medical Centers (VAMCs) to hire lung cancer screening program navigators. VA-PALS also provided resources for these nascent lung cancer screening programs to support navigators including trainings, monthly navigator roundtable meetings, in-person conferences, and access to lung cancer screening experts.^11,12^

Current evidence supports the effectiveness of navigators largely in the areas of cancer care, care for vulnerable or disadvantaged populations, and care for those receiving assistance for complex, chronic diseases.^13–15^ Despite evidence supporting navigator impact on clinical improvement, there remains a knowledge gap in understanding *how* navigators improve care and how best to design systems to effectively utilize navigators. As a part of the VA-PALS Program Evaluation, we performed a qualitative evaluation of the factors influencing the lung cancer screening program navigator role by interviewing key personnel at the 10 participating VAMCs.^11^ Guided by the health systems science framework and relational coordination theory, we elicited the perspectives of a diverse, national population of VHA healthcare team members involved in the development of lung cancer screening programs.^16–18^ We aimed to explore factors influencing the lung cancer screening program navigator role and the navigator’s ability to impact institutional program outcomes.

## METHODS

### Study Design and Setting

From December 2020 to September 2021, we conducted a cross-sectional qualitative study with a diverse sample of VHA healthcare team members involved in lung cancer screening at the 10 initial VA-PALS sites (Atlanta, Cleveland, Denver, Chicago-Hines, Indianapolis, Milwaukee, Nashville, Philadelphia, Phoenix, and St. Louis). All sites were considered by VHA to be a class 1a– or 1b–level facility by complexity score. VAMC complexity score consists of five complexity levels: 1a, 1b, 1c, 2, and 3, where 1a is the most complex and 3 is the least complex. This ranking system takes the following into consideration: (1) volume and patient case mix, (2) clinical services provided, (3) patient risk calculated from VA patient diagnosis, (4) total resident slots, (5) an index of multiple residency programs at a single facility, (6) total amount of research dollars, and (7) the number of specialized clinical services.^19^

The research team was located at the VA Tennessee Valley Healthcare System (TVHS) in Nashville, TN. This study was approved by both the VA Central Institutional Review Board (C-IRB E19-05) and the VA Tennessee Valley Healthcare System Research & Development Committee. The study was also approved by the VA Organizational Assessment Subcommittee and the VA Office of Labor and Management Relations.

### Study Participants and Recruitment

Potential interview participants included VHA healthcare team members (physicians in medical oncology, primary care, pulmonary, radiology, and surgery; advanced practice providers; nurses; radiology technicians; and administrators) identified by each site’s lung cancer screening program leadership. Some of these identified healthcare team members were directly involved with program operation and leadership, while others were involved in a referral or support capacity. We recruited potential participants via email, faculty and staff meetings, and professional contacts. Study team personnel contacted those who indicated interest in participating in in-depth interviews and obtained informed consent via telephone. Study personnel then scheduled the participant for a 1-h telephone interview. Participation incentives were not offered. Recruitment was stopped once data saturation was reached and no new themes were identified.^20^

### Theoretical Frameworks

The parent study VA-PALS Program Evaluation measures were guided by the RE-AIM (reach, effectiveness, adoption, implementation, and maintenance) framework.^21^ The semi-structured interview guide for this qualitative study was developed with the guidance of the consolidated framework for implementation research (CFIR) and health systems science framework a priori.^16,22^ We sought to explore broad contextual factors and to understand each program’s organizational structure and processes that may influence the lung cancer screening program navigator role. Health systems science core functional domains include healthcare structures and processes, health system improvement, healthcare value, population health, technology, and healthcare policy and economics.^16^

As themes emerged during data analysis, we recognized the importance of the navigator in facilitating the complex process of lung cancer screening. We further leveraged the relational coordination theory as a framework to explore the navigator’s role in the lung cancer screening process.^17,18^ Relational coordination theory proposes that relationships of shared knowledge, shared goals, and mutual respect can support timely, frequent, and accurate problem solving to positively influence desired outcomes. The three main components of the relational coordination theory include relational coordination as a process for coordinating work, the relational organizational structures that support this coordination, and the resulting outcomes.^17,18^

### Interview Guide

The semi-structured interview guide was tested, refined, and finalized by conducting pilot interviews with two providers (see Appendix A). The interview guide included questions about employment characteristics (employment history, percentage of clinical practice living in rural areas, roles and responsibilities), attitudes and beliefs about lung cancer screening, protocols and processes, barriers and facilitators to implementation, teamwork, and interprofessional communication.

Additional questions focused on navigators. In this report, we use the term “navigator” to refer to the person who facilitates the program processes by both providing direct patient care and coordinating program logistics. We also probed regarding program team characteristics, leadership buy-in, navigator role on the team, and navigator characteristics including work length, retention, and permanency. The questions were presented in a hypothetical frame for those participants at medical centers that had not yet established a lung cancer screening program.

### Data Collection and Analysis

The Vanderbilt University Qualitative Research Core (VU-QRC) managed data collection, coding, and analysis and reported following the Consolidated Criteria for Reporting Qualitative Research (COREQ) guidelines.^23^ A trained moderator (K.B., MA in Social Psychology, VU-QRC Senior Research Manager, female, 12 years’ experience, no prior relationship with study participants) conducted the telephone interviews using the interview guide. Follow-up questions were asked for clarifying purposes and to facilitate detailed discussion. Interviews lasted approximately 1 h. Participants knew the purpose of the study and that the interviewer was a trained qualitative researcher with no clinical background. We recorded interviews via a secure audio recording device with participant permission and transferred each recording to a secure, protected research folder, assigned a unique ID number, transcribed, and de-identified. Study personnel maintained a participant identification log on a password-protected server housed at the VA TVHS.

We developed a hierarchical coding system and refined it using the interview guides, review of the transcripts, and CFIR and the health systems science frameworks.^16,22^ Once themes emerged, we additionally used the relational coordination theory.^17,18^ Definitions and rules were written for the use of coding categories. Major categories are listed in Appendix B and were further divided from subcategories, with some subcategories having additional levels of hierarchical division. Categories included (1) screening activity; (2) lung cancer screening program characteristics; (3) hospital organizational setting; (4) outer setting; (5) individual characteristics; (6) patient factors; (7) communication; (8) barriers and facilitators; (9) specific examples; (10) process; (11) suggestions and needs; (12) provider/health team member; (13) practice/work experience; (14) world events; (15) change over time; (16) notable quotes; and (17) strategies and solutions.

Three experienced VU-QRC research assistants first established reliability in using the coding system on two transcripts. Coding of each transcript was compared, reconciling any discrepancies through discussion sections. After consensus was reached, the coders divided and independently coded the remaining transcripts. Each speaking turn was treated as a separate quote and could be assigned up to 24 distinct codes. The coded transcripts were combined and sorted by code. Transcripts, quotations, and codes were managed using Microsoft Excel 2016 and SPSS version 29.0.

### Development of a Framework for the Factors Influencing the Lung Cancer Screening Program Navigator Role

We used an iterative inductive/deductive approach to qualitative data analysis^24,25^ to develop our conceptual framework. Inductively, we sorted the coded quotes by category to identify higher order themes and relationships between themes.^16^ Deductively, we were guided by the health systems science framework, and later, as themes emerged, by the relational coordination theory.^17,18^ We were also guided by the knowledge of existing lung cancer screening program processes.^3^ The framework development process was iterative in that the conceptual framework is theoretically informed, while the specific content in the framework is derived inductively from the qualitative data.

**Figure 1:**
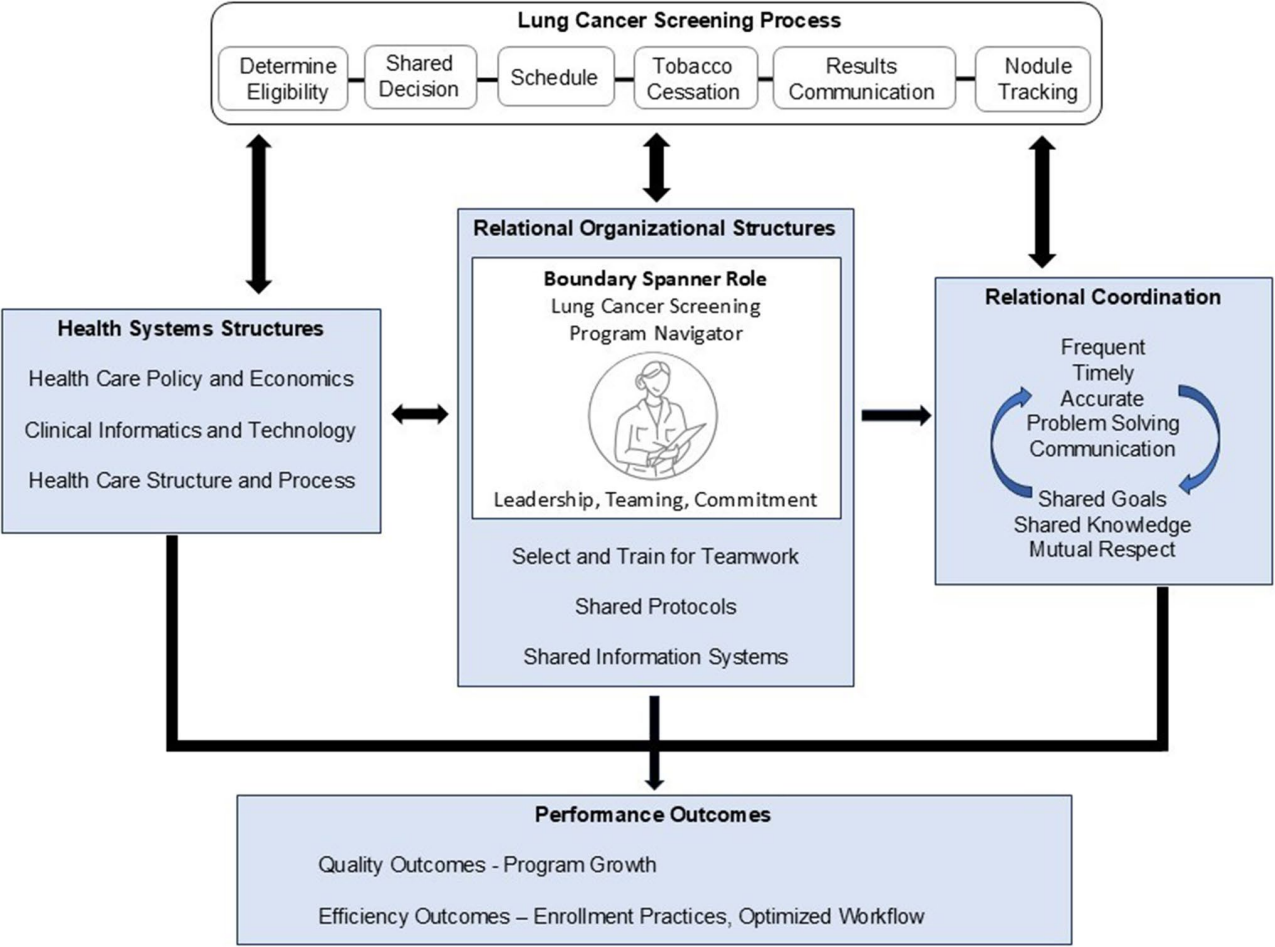
Conceptual framework of factors influencing the lung cancer screening program navigator role. The conceptual framework was developed inductively and deductively from in-depth interviews (*n* = 30) with a national sample of healthcare team members using the health systems science framework and relational coordination theory.^16–18^

Figure 1 depicts our framework, adapted from the health systems science framework and relational coordination theory, that describes factors influencing the lung cancer screening program navigator role.^16–18^ Factors include the lung cancer screening process, organizational structures adapted from the health systems science framework, and relational factors adapted from the relational coordination theory. The representation of the navigator in a boundary spanner role is central to the framework.

## RESULTS

### Participant Enrollment and Characteristics

We contacted 54 healthcare team members to ask if they would like to participate in an interview. Of these, 30 participants completed an interview (participation rate = 56%). Data from these 30 interviews is included in the final analytic sample. Table 1 describes the participant demographics.

**Table 1:** Participant Demographics

Characteristic	*n* (%) *N* = 30
VA site	
Atlanta	1 (3)
Chicago-Hines	2 (7)
Cleveland	3 (10)
Denver	3 (10)
Indianapolis	3 (10)
Milwaukee	3 (10)
Nashville	3 (10)
Philadelphia	5 (17)
Phoenix	3 (10)
St. Louis	4 (13)
Leadership role	
Lung cancer screening program director	10 (33)
Specialty	
Oncology	2 (7)
Primary care	10 (33)
Pulmonology	7 (23)
Radiology	7 (23)
Other or not applicable*	4 (13)
Professional training	
Physician	14 (47)
Nurse	6 (20)
Technician/technologist	4 (13)
Advanced practice provider	3 (10)
Administrative support assistant	2 (7)
Other**	1 (3)
Time in role	
1–10 years	11 (37)
11–20 years	10 (33)
> 20 years	9 (30)

^*^Some participants were in tobacco cessation outside of primary care, radiology, oncology, or pulmonology or in a medical support assistant role that did not align with a particular specialty

^**^One participant was in a program data analyst role

### Thematic Analysis

#### Health Systems Science: Organizational Structural Influences on Program Navigators

Table 2 presents supporting quotations denoted by themes and subthemes of the health systems science framework, with participant role indicated.^16^ Qualitative analysis identified several prominent organizational structural themes that influence the program navigator role, including healthcare structure and process, clinical informatics and technology, and healthcare policy and economics. It should be noted that some participants used the term “program coordinator” interchangeably with “program navigator.”

**Table 2:** Sample Quotations — Health Systems Science Domains

Healthcare structure and process	
Staffing	…if you don’t have that personnel (navigators) you are dead in the water. You cannot run the program. **Participant 24, program director, pulmonologist** If we had a critical mass where we could dedicate one person to set aside to do lung cancer screenings, then that would probably help with that issue. **Participant 26, radiologist** Four days a week dedicated to the screening program. She has one where she actually staffs the lung nodule management clinic with me and two other providers. But ironically those are people that are referred to our program from hers. People she’s identified. It’s sort of a self-fulling clinic. She’ll screen people and people who have nodules that need to be managed, we transfer them to the lung nodule program or the pulmonary nodule clinic. So, she’s actually, on occasion, seen people that she’s actually screened and identified with nodules which is kind of fun. So, it’s four days a week dedicated to the screening program and one day for pulmonary nodule management. **Participant 16, program director, pulmonologist**
Setting	Three years ago, we turned our lung cancer screening clinic into a telephone clinic and my lung cancer screening coordinator* works most of the time from home. They have a VA laptop with access to medical records and imaging and all the information and so I don’t need office space or parking for my lung cancer screening coordinators*. It’s a virtual clinic. Patients love it and we never bring a patient to the hospital to have shared decision making or to discuss results of the imaging. **Participant 24, program director, pulmonologist** So flexibility within the screening schedule to say okay Mr. Smith who lives 1.5 h away is going to be at the facility for another appointment, let’s do his shared decision making and his scan on this date or let’s reach out to him and do a telephone or a video shared decision making and say okay let’s coordinate and have you CT scan the day you’re coming to see your primary care or rheumatologist… **Participant 16, program director, pulmonologist**
Clinical informatics and technology	
	If you don’t have a system like that your computer integrated with the medical record to manage lung cancer screening, you’re going to be overwhelmed and your lung cancer screening coordinator*s are going to be stressed out and burned out and they’re going to quit and you’re going to be having a lot of turnover of your staff and then you’re going to be in trouble. **Participant 24, program director, pulmonologist** We’ve made an order set in CPRS so that when the nurse practitioners are done, they just literally have to click, click, click here’s what we want. We have protocolized everything and we have a lot of the templates for the letters for the appointment itself. So, we try to make everything as streamlined as possible. **Participant 19, program director, pulmonologist** So, we set up a system where when a person goes into order a CT scan in the radiology menu it says is this a lung cancer screening study? If you click yes, it sends an automatic sort of, it’s not a consult but it’s like a reminder or clinical alert to our nodule navigator. Our nodule navigator then calls the patient to make sure they’re eligible based on age, pack years and does the shared decision making. So that was a good fix because one it saves radiology an unnecessary procedure and two it helps us gather many of these people who are being screened. **Participant 17, program director, pulmonologist**
Healthcare policy and economics	
	Well, there are certainly competing priorities, right? So, you’re asking for them to bring on this provider to do this and again it’s not a mandated thing like colonoscopy or mammography, etc. So that was a challenge particularly at the VA during budgetary times where they’re fairly tight. **Participant 17, program director, pulmonologist** I think it helps also that initially her salary was paid for by VA-PALS and then through that because it was initially funded through the Office of Rural Health, it was done as a permanent position and then the hospital was able to take over funding her position…when we tried to get a coordinator* before that, it was very difficult, and we weren’t successful until the VA-PALS experience. **Participant 18, program director, oncologist**

^*^Although VA-PALS provided funding for program navigators who provided direct clinical care at all sites, some interview participants refer to these individuals interchangeably as “coordinators”

##### Healthcare Structure and Process

Study participants regularly identified staffing and setting (including lung cancer screening processes) as key healthcare structures that may influence a navigator’s role.

##### Staffing

Participants explicitly recognized the navigator’s central position in the screening program. Participants discussed the multiple efforts assigned to navigators. Most perceived navigators as dedicating a substantial level of effort to the lung cancer screening program. The absence of a navigator was perceived to be a risk that could jeopardize the program, leading to instability and vulnerability to error, with one interviewee saying “if you don’t have that personnel, you are dead in the water. You cannot run the program.”

##### Setting

Participants described program activities taking place in a variety of practice settings. Navigators often aligned program activities with patient needs and preferences by providing care at both hospital and community locations as well as offering telehealth options to Veterans, with a participant saying navigators can “reach out … and do a telephone or a video shared decision making and say okay let’s coordinate and have your CT scan the day you’re coming to see your primary care or rheumatologist.”

##### Clinical Informatics and Technology

Participants indicated the importance of administrative and communication infrastructure including electronic and computer systems resources for lung cancer screening program management. This included the importance of integrated computer systems that manage program data for navigator task facilitation. One participant stated, “If you don’t have a system like that your computer integrated with the medical record to manage lung cancer screening, you’re going to be overwhelmed.” Participants described the importance of program-specific electronic health record order sets and protocolized templates to simplify lung cancer screening processes. Automated communication systems were also reported as important to facilitate the clinical workflow and identification of eligible individuals to enroll in the screening program.

##### Healthcare Policy and Economics

Participants discussed the financial support needed for appropriate program navigator staffing. Numerous discussions of costs were accompanied by describing the initial economic constraints in establishing a permanent navigator position. One individual noted, “it helps … that initially her salary was paid for by VA-PALS …when we tried to get a coordinator before that, it was very difficult, and we weren’t successful until the VA-PALS experience.”

#### Relational Coordination Theory: Relational Influences on Program Navigators

Table 3 presents supporting quotations denoted by themes and subthemes of the relational coordination theory, with participant role indicated.^17,18^ Qualitative analysis identified several prominent relational themes that influence the program navigator role including organizational structures (boundary spanner role, select and train for teamwork, shared protocols and information systems), relational coordination, and performance outcomes. It should again be noted that some participants used the term “program coordinator” interchangeably with “program navigator.”

**Table 3:** Sample Quotations — Relational Coordination Theory Domains

Organizational structures	
Boundary spanner role	
Professional background	Well, she was in our area before and was one of our nurse partners, so I’d worked with her closely for about six years. I knew that if I gave her a task that it was going to get done. So, you want someone with a lot of VA experience that knows how to navigate it, but I also knew that she knew how to do stuff. **Participant 20, program director, oncologist** I tell them to call me anytime if you have questions. You know, they’re nurse practitioners, and they have training to know what’s urgent and what’s not. I mean none of these are typically very, very urgent but if it’s something like ‘hey I’m seeing this person later and I noticed that they have prostate cancer, but it looks like its localized, can I call you and see if this person would be a candidate for screening? **Participant 19, program director, pulmonologist**
Foundational skills	
Leadership	So, the most important person in the team is the program navigator. **Participant 24, program director, pulmonologist**
Teaming	I love working with her (navigator). We have a great communication. You know if something needs to be changed, she can call me and say, ‘I’ve got another person that’s just walked in’ or ‘can you change this person or overbook this person’? We work together as a team, and I really like that. And you know if this patient wants to speak with somebody, I can always Teams her and say, ‘look the patient wants to talk with someone now’ and she’ll say, ‘oh great I’ll call them now’… **Participant 23, medical support assistant**
Commitment	…she (navigator) does a presentation to this primary care group or a presentation to that primary care group to show them what the program is all about and why it’s going to be helpful and why they should do it. So, I think the more you can provide educational resources or individuals the easier you’re going to spread the word. **Participant 20, program director, oncologist** We have had, especially since our coordinator* started, she has done outreach to the rural providers in the outlying CBOCs to talk about lung screening. **Participant 18, program director, oncologist**
Select and train for teamwork	I trained my nurses on all the steps of the processes of lung cancer screening. I trained my nurses on how to communicate with the Veterans that are enrolled in the programs or canvassed to be enrolled. How to communicate results and then how to document all of this in the medical record so that I can review and sign all these clinic notes and orders for images. **Participant 24, pulmonologist, program director** Well, the idea is to train the nurse practitioners so that they can take care of almost everything on a day-to-day basis. So, and to be honest, I have protocols for the shared decision making, I have protocols on the follow up and I have protocols for our data management system. And so, a lot of that is education up front and of course the communication is more early on…There’s a lot early on and a lot of hands on and personally being there and shadowing, showing here’s how you call, and this is what it looks like and here’s what the conversation is and here’s how we document. **Participant 19, program director, pulmonologist**
Shared protocols and information systems	So, I think we all, the main two people who are involved in the program are myself and the nurse practitioner/lung cancer screening coordinator. So, I think we have kind of a shared responsibility in that since we meet every week with the lead lung cancer screening coordinator at VISN (location) who helps implement these programs around the country. So, we have a weekly meeting with her where we bring up issues that we’re having. So, I think it’s a shared responsibility between us and VISN (location) helps out and then we have a steering committee that generally meets every one to two months depending on schedule where we have different stakeholders who also have a role, so oncology, radiology, primary care and pulmonary. So, I think it’s a shared responsibility but most of it falls, day to day to (Navigator), the nurse practitioner and myself. **Participant 14, program director, pulmonologist** So, I think we all, the main two people who are involved in the program are myself and the nurse practitioner/lung cancer screening coordinator*. So, I think we have kind of a shared responsibility in that since we meet every week with the lead lung cancer screening coordinator* at VISN (location) who helps implement these programs around the country. So, we have a weekly meeting with her where we bring up issues that we’re having. So, I think it’s a shared responsibility between us and VISN (location) helps out and then we have a steering committee that generally meets every one to two months depending on schedule where we have different stakeholders who also have a role, so oncology, radiology, primary care and pulmonary. So, I think it’s a shared responsibility but most of it falls, day to day to (Navigator), the nurse practitioner and myself. **Participant 14, program director, pulmonologist** The pulmonary and the radiology clinicians need to work with the lung cancer screening coordinator* shoulder to shoulder every day and need to have meetings every week to discuss difficult or peculiar cases that may need a different approach…everybody needs to be following and using the same protocol so that everybody is managing the patients the same. That’s pretty much the team. That’s what you need. **Participant 24, program director, pulmonologist**
Relational coordination	
	…when you communicate with shared decision making, you’re making sure that you are speaking to them in terms that they understand and can relate to and can appreciate and that you’re not really speaking over them or giving complex concepts or thoughts that they may not really understand. So, to me that’s one of the biggest things. Our navigator does an amazing job just bringing the shared decision making down to earth, just making it very easy to understand, and making the Veterans feel comfortable that they can approach and ask questions. **Participant 16, program director, pulmonology** …so (Navigator) is really the heart of the operation. This is one of many things I’m kind of overseeing so I really need my other players to kind of run things. So (Navigator) is the one that really has set up our program and is expanding. Ultimately, we will need to hire a second person because she won’t be able to do it all. **Participant 20, program director, oncologist**
Performance outcomes	
Quality	You know we had (prior to Navigator) the smoking cessation, we were doing the shared decision making, we were doing some tracking, but we really didn’t have the cohesiveness and the dedicated resource to do it how we wanted to do it. Bottom line through the VA-PALS, we were able to hire a nurse practitioner who now is the main person. So, all the primary care doctors who are asking the patient and has deemed the patient to be eligible, they place the order, all the referrals go through our lung cancer screening coordinator* who’s a nurse practitioner. She does the shared decision making, refers the patients to smoking cessation, tracks the nodules, and completes the visit either through e-consult or VVC which has certainly been a good feature through the pandemic. So yes, that’s where we are today. We are able to enroll about 1400 patients in about maybe a year but less than a year and a half of being a VA-PALS center. And then we, if I’m not mistaken have identified 43 lung cancers with the lung cancer screening program which is the highest from all the VA-PALS centers. So, we are very proud. **Participant 15, program director, pulmonologist** You know we really have done thousands of patients already and that’s a huge job to keep up with to make sure all the data is entered and tracked appropriately. And it was tough enough to get the one coordinator*, it took us a long time, but I can easily see our volume continuing to increase to the point where we’re going to need another coordinator* and getting that resource in place will be a challenge… **Participant 2, radiologist** when you get big enough you also need to have the support. One navigator should only manage, I don’t know, a thousand patients. If there’s some finite level that one person can reasonably manage so as we get bigger, we have to be more efficient to manage more patients and then we have to be staffed appropriately. **Participant 17, program director, pulmonologist**
Efficiency	I say initially the primary care providers were doing the screening assessment and now that we have a coordinator* on board that role really falls to the coordinator* to make sure that people are in fact eligible. **Participant 2, radiologist** I think what’s helpful for us is that (Navigator), our nurse practitioner that kind of heads that program, she orders the exams herself. So that’s helpful when we see her name and even if its ordered as a thorax without because I believe again there may be like insurance issue where that lung cancer screening order may only be able to be scanned like once a year for insurance purposes but then we perform and its scanned as a thorax without but we still use the lung cancer screening protocol if you can understand that. So, there’s different series that are included for the lung cancer screening like I said before. There’s like slimmer slices. So, the order per se itself will say thorax without but when we see its from (Navigator) and she’ll put lung cancer screening three month follow up or six month follow, we know we have to scan it as a lung cancer screening exam… **Participant 22, CT supervisor** I would say the major limitation for us was really radiology. We did have a Chief of Radiology who was quite not opposed to it in the real sense, but really worried about the workload that this would pose on his department and the volume and the resources that would be needed. So, for me, the most amount of convincing was really with him. And really for him understanding that they didn’t really have to look at, if we would go to the lung cancer screening coordinator*, he wouldn’t have to look at the lung cancer screening criteria. All those scans would be ordered properly. You wouldn’t order a lung cancer screening scan on a patient that had previous nodules or has history of lung cancer so that didn’t have to spend time on that because we would do that. I think that was probably reassuring to him. **Participant 15, program director, pulmonologist** I’d say the most difficult thing was getting the required personnel onboard that would be able to logistically conduct the shared decision making and the screening. Once we had the designated nurse practitioner, we had very good collaboration with primary care and they were very willing to submit the consults for the lung cancer screening program… **Participant 16, program director, pulmonology**

^*^Although VA-PALS provided funding for program navigators who provided direct clinical care at all sites, some interview participants refer to these individuals interchangeably as “coordinators”

**Organizational Structures**

##### Boundary Spanner Role

Respondents repeatedly describe the navigator as someone who is at the epicenter of a program, working directly with patients, but also with a multidisciplinary team of clinicians including primary care, pulmonology, surgery, and radiology. One individual stated, “the most important person in the team is the program navigator.” In this respect, the navigator serves in a *boundary spanner role* which is defined by the relational coordination theory as a job that is designed to bridge work across organizational boundaries and support the development of relational coordination.

Interviewees described several navigator personal attributes and characteristics such as professional background and foundational skills that likely contribute to a navigator’s impact in this boundary spanner role. (1) Professional background — the navigator role was often filled by registered nurses and advanced practice providers. Prior clinical experience was deemed crucial for independent decision-making and an ability to function autonomously in unique and interdisciplinary clinical environments. These characteristics were highly favored by lung cancer screening program directors. (2) Foundational skills — leadership, teaming, and program commitment were identified as key foundational skills for successful management of a lung cancer screening program.

-Leadership. Effective leadership skills promoted team motivation and maintenance of a shared vision and goals for lung cancer screening programs. The interdisciplinary structure of the lung cancer screening programs provided opportunity for navigators to become leaders at each site’s program steering committee level.
-Teaming. Site program directors highlighted the structured and collaborative practices of navigators as fundamental to ensure adherence to lung cancer screening recommendations. Navigators and site program directors shared the responsibility of relaying organizational recommendations to the full lung cancer screening program teams at their local site. Participants recognized the importance of a navigator’s ability to work in the team setting. One interviewee stated, “we work together as a team, and I really like that.”
-Commitment. Navigators demonstrated commitment to their lung cancer screening program by seeking opportunities to lead program outreach efforts. They were also recognized for their work in educational outreach to providers in rural settings within their organization.

##### Select and Train for Teamwork

Many participants described the comprehensive navigator training designed to learn and share experiences about navigator roles and responsibilities. This training can prepare navigators for effective communication, documentation requirements, shared decision-making, and population health data management systems. One interview participant said, “Well, the idea is to train the nurse practitioners so that they can take care of almost everything on a day-to-day basis.” It was acknowledged that such training helped accelerate navigator autonomy, ability to complete tasks independently, and ability to effectively communicate with patients and the healthcare team.

##### Shared Protocols and Information Systems

Interviewees reinforced the importance of shared protocols and information systems, not only within individual programs, but across programs to share best practices and learned experiences, with one participant stating, “everybody needs to be following and using the same protocol so that everybody is managing the patients the same.”

##### Relational Coordination

Participants overwhelmingly support the navigator’s key role in relational coordination, the mutually reinforcing process of communicating with a purpose of task integration and improved outcomes. Navigators participate in frequent, timely communication with both patients and the lung screening clinical care team. One interviewee stated, “when you communicate with shared decision making, you’re making sure that you are speaking to them in terms that they understand.” Interviewees suggest navigators are central to lung cancer screening program relational coordination, saying “(Navigator) is really the heart of the operation… (Navigator) is the one that really has set up our program and is expanding. Ultimately, we will need to hire a second person because she won’t be able to do it all.”

##### Performance Outcomes

Interview participants directly linked their VAMC’s lung cancer screening program outcomes to the navigator. Program performance outcomes specifically attributed to the navigator role include (1) quality outcomes (program growth) and (2) efficiency (enrollment practices and optimized workflow).

##### Quality

Participants attributed lung cancer screening program growth to navigators. Navigators were described as the “heart” of the program and the source of program expansion. As programs continue to expand, participants anticipated the need to advocate and justify additional navigator positions. Multiple navigators may be needed to ensure quality data management and patient satisfaction. One participant stated, “we really have done thousands of patients already and that’s a huge job to keep up with to make sure all the data is entered and tracked appropriately. And it was tough enough to get the one coordinator, it took us a long time, but I can easily see our volume continuing to increase to the point where we’re going to need another coordinator.”

##### Efficiency

Proper patient selection, quality of shared decision-making, and clearly defined personnel roles were identified as efficient enrollment practices. Participants described the navigators’ ability to determine patient eligibility as a factor contributing to provider efficiency, stating “initially the primary care providers were doing the screening assessment and now that we have a coordinator on board that role really falls to the coordinator to make sure that people are in fact eligible.” Participants further described observing lung cancer screening hesitancy from among both primary care providers and institutional leaders. This hesitancy was believed to be due to anticipated clinical workflow challenges for busy practitioners. Interview participants noted that navigator capabilities and responsibilities can alleviate many of these concerns. For example, shared decision-making, when properly performed, can take a considerable amount of time, leading to workflow “bottlenecks” within the primary care clinical visit, where there is often limited time to cover many topics. Participants indicated an uptake in consultations placed by primary care after introducing a navigator able to conduct shared decision-making, with one participant saying, “the most difficult thing was getting the required personnel onboard … to logistically conduct the shared decision making and the screening. Once we had the designated nurse practitioner, we had very good collaboration with primary care, and they were very willing to submit the consults for the lung cancer screening program.”

## DISCUSSION

Healthcare team members involved in lung cancer screening nationally across the VHA identified several structural and relational factors influencing both the navigator role and the navigator’s ability to influence program outcomes. Specifically, the importance of relational coordination and the program navigator’s role as a boundary spanner role emerged as key themes. Healthcare team members identified certain foundational navigator attributes including leadership, teaming, and commitment as key to facilitating the navigator’s boundary spanner role in the lung cancer screening process. Effective leadership was described as promoting team motivation and the maintenance of a shared vision and goals for lung cancer screening program growth and effectiveness. Teaming, in the form of teamwork and interprofessional communication skills, was described as imperative to improving lung cancer screening program outcomes. Navigators with teaming skills were described as recognizing the expertise of the interprofessional team. Navigators demonstrating commitment were noted to seek opportunities to lead program growth and outreach efforts. Overall, healthcare team members identified the navigator as a critical role in the lung cancer screening program, with participants stating, “if you don’t have that personnel, you are dead in the water. You cannot run the program” and “the most important person in the team is the program navigator.”

Our exploration of the lung cancer screening program navigator role aligns with the relational coordination theory, which proposes that coordination (the management of interdependent tasks) that occurs through high-quality, frequent communication, mutual respect, shared knowledge, and shared goals helps healthcare organizations to achieve their desired outcomes.^17,18^ In our study, healthcare team members identified lung cancer screening program navigators as a key component in developing relationships and facilitating frequent, high-quality communication. Organizations which provide structural resources (salary support, clinical informatics, technology) in addition to relational resources (training, shared protocols, support for navigator leadership) have an opportunity to support the navigator to positively influence program outcomes of quality and efficiency.

Our findings support previous work demonstrating the complexity of the lung cancer screening process and the need for dedicated program resources.^26^ Navigators are one of the most critical resources designed to improve initial screening uptake, annual follow-up adherence, and patient satisfaction in the complex lung cancer screening process.^5,7,27^ Our qualitative evaluation of healthcare team members offers insight into the factors that may affect a navigator’s ability to impact program outcomes. As lung cancer screening programs grow and evolve, a more complete understanding of navigator attributes, and how institutions can support navigators, offers an opportunity for program improvement, evolution, and adaptation to meet additional challenges.^28,29^

This study is strengthened by the national sample of multidisciplinary healthcare team members at 10 VAMCs. This qualitative study followed rigorous COREQ guidelines and utilized several established implementation science frameworks to evaluate the overall program (RE-AIM, CFIR).^21–23^ Additionally, evaluation of the role of the navigator in the lung cancer screening leveraged and adapted two more detailed frameworks, the health systems science framework and relational coordination theory.^16–18^ Limitations should be noted. This qualitative evaluation may have the potential for selection biases due to recruitment primarily by word of mouth, leading to possible selection bias. Similarly, a large portion of the sampled healthcare team members held program leadership roles, which may have led to possible response and social desirability biases by participants. Participants were all selected from high complexity level VAMCs, which may potentially have more resources. The issues raised within this report may not reflect a broader selection of institutions that have fewer resources. The current study is also limited to the perspectives of healthcare team members only; patient perspectives were not elicited. Finally, this study was performed within the VHA healthcare system only, and caution should be used when generalizing to other healthcare settings.

## CONCLUSION

Healthcare team members involved in lung cancer screening indicate that several structural and relational factors influence the lung cancer screening program navigator role. While structural factors such as program setting, financial support, and technological resources influence the program navigator’s ability to impact outcomes, relational factors such as a navigator’s individual foundational skills (leadership, teaming, and commitment) and their role as a boundary spanner who crosses clinical silos facilitate a robust, effective, efficient lung cancer screening program. As lung cancer screening programs continue to evolve and grow, understanding and supporting the program navigator role provides an opportunity to improve lung cancer screening program outcomes.

## Supplementary Information

Below is the link to the electronic supplementary material.

Supplementary file1 (PDF 67 KB)

Supplementary file2 (PDF 84 KB)

## Data Availability

Data underlying this manuscript will be shared by the corresponding author upon reasonable request according to Department of Veterans Affairs policy.
