# Supplementary material for: A Qualitative Evaluation of Factors Influencing the Lung Cancer Screening Program Navigator Role
Source: J Gen Intern Med. 2025 Jul 25;40(13):3086–96. doi: 10.1007/s11606-025-09714-0 (PMC12508303; doi:10.1007/s11606-025-09714-0)
Supplement: Supplementary file 1 — Supplementary file1 (PDF 67 KB) [file 11606_2025_9714_MOESM1_ESM.pdf]

Project Title: Lung Cancer Screening in the VA

Method: In-depth Interviews

Target Audience: 10 interviews per site, 3 radiologists, 3 primary care providers (MD/APPs), 3 staff (plan to target primary care schedulers, radiology technicians), 1 administrative leader

Supplies: 4 recorders, batteries, 2 notepads, 6 pens, 14 consent forms

Total participant time required: 1.0 hr

Introduction (10 minutes)

Thank you for agreeing to an interview today. My name is _______ and I am from Nashville VA. We are conducting research on the implementation of low-dose computed tomography (low-dose CT) for lung cancer screening (LCS) at your VA. We feel that it is very important to speak directly to you about your experiences. Therefore, we will be conducting interviews with VA providers and staff in the areas of primary care and radiology.

During the interview today, we will be asking about your experiences and thoughts on cancer screening, focused primarily on lung cancer screening. We are most interested to hear about your own personal experiences, thoughts, and opinions of the new lung cancer screening test, low-dose CT. So, please do not feel shy. Your views are extremely valuable to us, and we are here to learn from you. I have a list of topics I would like to discuss with you, but feel free to bring up any topics you feel are related to the discussion. Also, I want to let you know that your participation in this interview is completely voluntary. If you want to stop at any time or don't feel comfortable answering a question, please let me know.

I would like to record our discussion so that the rest of the research team can also hear your views exactly and we don't miss anything you say. Our discussion will remain confidential; only the research team will listen to the recording and the information you give will only be used for this research project to improve lung cancer screening implementation in the VA. **Is it ok to record the discussion? Our interview will last about one hour. Are there any questions before we start? (complete consent form)**

Warm Up (10 minutes)

1. **How long have you been a ________(provider/staff/administrator) in _______ (specialty field)?**
2. **Where else have you practiced/worked in the past 5 years?**
   -*VA and non-VA*
3. **What percentage of your clinical practice live in a rural area?** (primary providers only) **Do you have a sense of how many Veterans at your VA live in rural areas?** (radiologists, staff/admin) (feel free to skip this question if many radiologists, staff/admin are not able to answer)
4. **Tell me about your thoughts on lung cancer screening?**
   Do you think it is worthwhile? Will it benefit patients? Is it valuable?
   Is there evidence that lung cancer screening saves lives from lung cancer (providers only)?

Is lung cancer screening a priority for you (primary care providers only)?
Is lung cancer screening a priority for your radiology service? (radiology providers only)

Main Questions (40 minutes)

(Place the page outlining the screening process down in front of participant and read it with them)

Lung Cancer Screening Involves the Following Activities:

1. Identification of patients who are appropriate for screening (older persons, heavy smokers)
2. Providers and patients discuss the benefits and risks of screening (shared decision-making)
3. Provider orders screening test or refers the patient for screening
4. Appointments for smoking cessation and screening are scheduled
5. Smoking cessation services offered
6. Screening study (CT scan) is performed
7. Radiologist interprets the screening study
8. Screening results are sent to the referring provider
9. Referring provider discusses the screening results with the patient
10. Results are tracked over time in an electronic software system
11. Annual screening follow up or further evaluation is scheduled

5. **Tell me about your role in lung cancer screening.**
   Were you involved in or are you currently involved in the decision to implement a lung cancer screening program at your VA?
   Describe the process your VA went through to start a lung screening program. If you are currently in the decision-making process, what considerations have come up?
   Who would be responsible for ensuring that the lung cancer screening is implemented correctly and consistently over time?

6. **What has been difficult in performing lung cancer screening?** (ask everyone)
- *Identifying eligible individuals, performing shared decision making (discussing potential risks/benefits) [who is performing the shared decision making with the veterans- any difficulties with that?], who shares the results with veterans- who is following up on screening results, knowing what next steps to take based on screening results [combo of staff and veteran], identifying those who need a repeat screening in 3,6, or 12 months; patients are not motivated, patients cannot afford screening, leadership does not support screening [service level or executive leadership level]*

7. **FOR PROGRAM DIRECTORS ONLY:** Tell me about the lung cancer screening program at your VA.
   - Who is on the team (specialties represented (i.e. radiology, pulmonology, primary care, oncology, surgery, etc.; types of providers such as physicians, nurse practitioners/physician assistants, or nurses)

- How many navigators are on the team? Have there been previous navigators? How many days a week do the navigator(s) work for the lung cancer screening program?
- Describe how the team works together on a daily basis? How does the team communicate? Handle challenges?
- How often does the team meet? Where? Was it more frequent meetings in the beginning?
- Do you interact with leaders at your VA on behalf of the lung cancer screening program? If so, how often? What type of communication (in-person meetings, emails, etc)? What is typically discussed?
- Do you interact with leaders at the VISN level? Does lung cancer screening seem like a priority of your VISN? If so, please describe.

8. **What has been difficult in implementing a lung cancer screening program?** (ask this question if they have been involved in implementing a program from #6 above)

   What do you think or anticipate would be some of the barriers to implementing a lung cancer screening program? (ask this question if they have NOT been involved in implementing a program from #6 above)
   - Do you have any experience in implementing a program in the VA? (if struggling to answer question)
   - Obtaining buy-in from primary care, creating a multidisciplinary team multiple, gaining buy-in from leadership, coordinating Veteran care, providing smoking cessation, [who offers the smoking cessation?], tracking screening results and who is screened, etc.

9. **What has been difficult in screening Veterans from rural areas? Do you know of any patient barriers that would make it difficult for screening Veterans from rural areas, at least from your clinical experience?**

   Patient barriers: reaching patients, fear, anxiety, financial costs, lack of awareness of screening, doesn't think it will help [e.g., trust that test is useful, trust in health system], transportation

   Healthcare system barriers: [I understand that a lot of steps are done for tools communication, electronic tools like telehealth] insurance, availability of follow up appointment times for the follow-up procedures and does your local VA offer that or do they need to go to another VA that offers that service; electronic tools, workflow, communication, appointment time [what about rural provider awareness- word without sounding insulting]

10. **FOR PROGRAM DIRECTORS ONLY**: Is the navigator in your program a permanent position? Have you had any challenges with making this position permanent? If yes, please describe (data will only be reported as aggregate and will be de-identified)

11. **What could help you perform lung cancer screening?** --- only for providers

    What kinds of changes in the current support system could help you? --- for staff/admin

    What do you think could help screen rural Veterans? (ask separately)

- *[start broad and then go to rural] Provider level: educational materials – describe type website, posters, handouts, video for waiting room,*
- *[helpful to have Edu materials to providers or patients- Provider: If lecture, describe preferences on amount of time and type of lecture – webinar, grand rounds, multidisciplinary, CME. application for handheld electronic, lecture.*
- *provider/patient reminder systems (describe preferences) how long should it be and how often should it be offered?*
- *System level: changes in information systems or electronic records systems, leadership, policies, other?*
- *[any difference for rural area] unique to the rural- talk about a lot of barriers in general to lcs-[make not should redundant- eg. We've talked a lot about barriers specific to rural and are any other barriers that are unique/specific to the rural population?]*

12. **How well does lung cancer screening fit with the existing work processes and practices in your VA?** *---only for providers*
    - *What are likely issues or complications that may arise? [e.g. streamlining and integrated into workflow]*

13. **Are meetings held regularly to discuss work processes and practices such as lung cancer screening?**
    - Do you have a steering committee or regular meetings to discuss how things are going with lung cancer screening? [interested if other sites have a steering committee]

    If they know they have a steering committee then ask:
    Do you typically attend?
    Do you know who typically attends these meetings?
    What proportion of those expected to attend typically attend?
    How often are the meetings held?
    What is a typical agenda? How helpful are these meetings?

14. **What is currently working well with lung cancer screening at your VA? What is working well for screening Veterans who live in rural areas? What could work better? [stay with broad until ready to specific to rural] –** only for staff/admin
    a. *<u>(staff/administrators question probe)</u>*
        - *workflow, coordination, communication, increase in demand/radiology appointments, smoking history availability in chart, smoking cessation, shared decision-making documentation*

15. **What are your hopes for the future of lung cancer screening? What could help maintain the program?** *<u>(administrators)</u>* – only for staff/admin
    -provide excellent medical care to Veterans, cost effectiveness, team-based approach to providing high-quality care

<u>As a non-MD (or someone who is not involved in direct patient care) is there anything I did not think to ask that you think would be helpful</u>

Thank you very much for sharing your thoughts and experiences with us. We are learning a lot about this new screening test and your perspective is incredibly valuable as we aim to improve lung cancer screening implementation in the VA. We will provide you with lunch as a token of our appreciation.
