# Supplementary material for: A Qualitative Evaluation of Factors Influencing the Lung Cancer Screening Program Navigator Role
Source: J Gen Intern Med. 2025 Jul 25;40(13):3086–96. doi: 10.1007/s11606-025-09714-0 (PMC12508303; doi:10.1007/s11606-025-09714-0)
Supplement: Supplementary file 2 — Supplementary file2 (PDF 84 KB) [file 11606_2025_9714_MOESM2_ESM.pdf]

Appendix B. Coding Categories	
**Categories/Subcategories**	**Description**
**Screening Activity**	**Screening activity description**
Identify and initiate contact	Identification of patients who are appropriate for screening (older persons, heavy smokers) & following up with an outreach process
Shared decision making	Providers and patients discuss the benefits and risks of screening prior to making a decision (shared decision-making)
Test ordered	Provider orders screening test or refers the patient for screening
Smoking cessation offered	Smoking cessation services are offered
Coordinate, schedule appointment	Appointments for smoking cessation and screening are scheduled for the patient/s
Complete scan	Screening study (CT scan) is performed holistically, with adequate data collected
Radiology interpretation	Radiologist interprets the screening study & provides a comprehensive summary
Interpretation sent to referring provider	Screening results, along with data interpretation, are sent to the referring provider
Present results to patient	Referring provider discusses the screening results with the patient
Track and screen	Results are tracked over time in an electronic software system
Follow up	Annual or timely screening follow up or further evaluation is scheduled
Other screening activity	Other screening activity not listed above
**Lung cancer screening program characteristics**	**Discussion centers on characteristics of the lung cancer screening program**
Lung cancer screening program source	Discussion centers on how lung cancer screening program was developed (e.g., internally or externally)
Evidence Strength and Quality	Stakeholders' perceptions of the quality and validity of evidence supporting the belief that the lung cancer screening program will have desired outcomes.
Complexity	Perceived difficulty of implementation, reflected by duration, scope, radicalness, disruptiveness, centrality, and intricacy and number of steps required to implement.
Relative advantage	Stakeholders' perception of the advantage of implementing the lung cancer screening program versus an alternative solution.
Adaptability	The degree to which an lung cancer screening program can be adapted, tailored, refined, or reinvented to meet clinic or patient needs.
CT scan	Duration, structure, completeness
Features, structure	specific according to the intervention itself
Content data	specific according to the intervention itself
Cost	Discussion centers on costs associated with implementation
Trialability	The ability to test the intervention on a small scale in the organization, and to be able to reverse course (undo implementation) if warranted.
Other program characteristic	Other lung cancer screening program characteristic not listed above
**Hospital Organization setting**	**Discussion centers on clinic characteristics/Inner setting**
Structural characteristics	Clinic architecture, size, age, etc.
Clinic or organizational culture	Unique aspects of clinic (e.g., patient population, workflow, clinic norms/requirements/restrictions, protocols and processes)
Implementation climate	Discussion centers on capacity for change, shared receptivity of the lung cancer screening program, etc.
Compatibility	The degree of tangible fit between meaning and values attached to the lung cancer screening program by involved individuals, how those align with individuals' own norms, values, and perceived risks and needs, and how the lung cancer screening program fits with existing workflows and systems.
Relative priority	Individuals' shared perception of the importance of the implementation within the organization.
Available resources	Possible resources dedicated for specifically for implementation (e.g., funding, staff retention)
Access to knowledge and information	Ease of access to digestible information and knowledge about the lung cancer screening program and how to incorporate it into work tasks.
Other inner setting	Other hospital or organizational discussion not listed above
**Outer Setting**	**CFIR outer setting constructs**
Patient Needs and Resources	The extent to which patient needs, as well as barriers and facilitators to meet those needs, are accurately known and prioritized by the organization. (e.g., social determinants/disparities)
Cosmopolitanism	The degree to which an organization is networked with other external organizations (local and national/global)
Clinical Informatic, Health Information Technology	Focus on the degree to which application of data analytics and technology are utilized to improve organization characteristics (as it pertains to health information data)

Pressure to implement	Mimetic or competitive pressure to implement an lung cancer screening program; typically because most or other key peer or competing organizations have already implemented or are in a bid for a competitive edge.
Guidelines, recommendations	Knowledge/utilization of guidelines, recommendations, or other decision-making tools
Other outer setting	Other outer setting discussion not listed above
**Individual characteristics**	**Constructs related to behaviors, attitudes, beliefs, and motivation of clinical team**
Knowledge, familiarity with the facts	Knowledge and familiarity with the lung cancer screening program
Knowledgeable	understanding of situation at hand
Limited, insufficient	(note: some interviewees did not know what the lung cancer screening program was about, although was under implementation)
Organizational specific beliefs	Perceived behavioral expectations associated with the lung cancer screening program. Also includes perception of other people's behaviors associated with the problem or the lung cancer screening program.
Descriptive	Perception of the behaviors of other people around you; what people typically do
Injunctive	Behaviors one is perceived to be expected to perform and also expects others to perform
Control beliefs	The extent to which one's beliefs control themselves/to which one exhibits beliefs that are characteristic of exhibiting control in different areas
Self-efficacy	Ability to engage in the lung cancer screening program (e.g., belief in one's own ability to perform a behavior)
Individual stage of change	Stage of the individual (e.g., enthusiastic about lung cancer screening program)
Identification with organization	A broad construct related to how individuals perceive the organization, and their relationship and degree of commitment with that organization.
Program concerns	Specific concern related to the lung cancer screening program or implementation of the program
Anticipated outcomes	Discussion centers on participant's anticipated outcomes of the program (e.g.,likely issues or complications that may arise)
Causal attributions	Discussion centers on what the participant associates as a cause for a given problem, success, etc.
Social Comparison	Discussion centers on individual drive to gain accurate self, patient, or institutional evaluations. Also comparison to other screening types
Experience with similar programs	Discussion centers around an experience with a similar program(s)
Preferences	Any discussion associated with a greater liking to one option over another
Other individual characteristic	Other individual characteristic not listed above
**Patient factors**	**Discussion centers on specific patient factors**
Knowledge and understanding	Pt knowledge and understanding about lung cancer screening
Symptoms	Any physical or mental feature which is regarded as indicating a condition of disease, particularly such a feature that is apparent to the patient.
Co-morbidity	Discussion centers on the simultaneous presence of two or more diseases or medical conditions in a patient.
Health behaviors	Patient health behaviors
Healthy	Engages in healthy behaviors (e.g., healthy diet)
Not healthy, harmful	Engages in unhealthy behaviors (e.g., smoking)
Environment	Discusses patient's home/city environment, etc.
Rural	in, relating to, or characteristic of the countryside rather than the town.
Urban	in, relating to, or characteristic of a town or city.
Insurance coverage	Discussion centers on insurance status or coverage
Logistical factors	E.g., access to reliable transportation
SES, financial situation	socioeconomic status and other financial factors that influence the patient's financial status (income, family support, insurance, etc.)
Competing priorities	Priorities such as family engagement, limited time, etc.
Intrapersonal factors	e.g., motivation, self-efficacy, emotions and coping
Interpersonal factors	e.g., social support networks
Military service	Any discussion related to patient military service
Other patient factor	Other patient factor not listed above
**Communication**	**Discussion centering on the quality of communication**
Between providers	Discussion centers on communication between providers
Interprofessional engagement	Knowledge of interprofessional roles and responsibilities and utilizing the skills of the interprofessional team
Between patients and providers	Discussion focuses on the communication that occurs between patients and providers
Communication Modalities	Discussion centers on mode of communication; methods/information sources

In person	Discussion centers on in-person communication
EMR basketnote	Discussion centers on provider/interprofessional communication by way of EMR, notes, etc.
Telephone	Discussion between providers via phone
Telehealth	Discussion centers on patient communication through telehealth
Other communication modality	Discussion centers on other forms of communication
Goals and feedback	The degree to which program goals are clearly communicated, acted upon, and fed back to staff, and alignment of that feedback with goals.
Quality of communication	Any discussion related to the efficacy and clarity of communication
**Barriers Facilitators**	**Participant describes a potential challenges or facilitators to successfully implement the program**
Barriers, challenges	Any event/situation that lead to a breakdown in the process of program implementation and/or screening protocol.
Systems level	Any discussion of a challenge/barrier involved systemic level issues including but not limited to buy in, funding, interfacility communication
Patient level	Any challenge/barrier involved patient issues including but not limited to anxiety, transport, education etc.
Facilitators	Any discussion of factors that enabled program implementation and/or screening protocol success
Contingency conditional	Participant's response is contingent on a given factor
**Specific examples**	**Specific examples of situations/instances**
Went, going well	program implementation was successful/is going well
Error of omission	Program implementations that were excluded or unsuccessful
Error of commission	Program implementations unsuccessful due to implementation or screening errors
General example	Example not specified as went well/did not go well
Not observed	Participant states that s/he as not experienced a specific topic
**Process**	**Describe the process your VA went through to start a lung screening program. If you are currently in the decision-making process, what considerations have come up?**
Planning	The degree to which a scheme or method of behavior and tasks for implementing an lung cancer screening program are developed in advance, and the quality of those schemes or methods.
Engaging	Attracting and involving appropriate individuals in the implementation and use of the lung cancer screening program through a combined strategy of social marketing, education, role modeling, training, and other similar activities.
Opinion leaders	Individuals in an organization who have formal or informal influence on the attitudes and beliefs of their colleagues with respect to implementing the lung cancer screening program.
Formally appointed internal implementation leaders	Individuals from within the organization who have been formally appointed with responsibility for implementing an lung cancer screening program as nurse navigator, coordinator, project manager, team leader, or other similar role.
External change agents	Individuals who are affiliated with an outside entity who formally influence or facilitate lung cancer screening program decisions in a desirable direction.
Executing	Carrying out or accomplishing the implementation according to plan.
Reflecting and evaluating	Quantitative and qualitative feedback about the progress and quality of implementation accompanied with regular personal and team debriefing about progress and experience.
**Suggestions and Needs**	**What kinds of changes in the current support system could help you? (will likely co-occur with other codes)**
Systems level	Systems-level suggestions and needs (e.g., changes in information systems or electronic records systems, leadership, policies)
Patient level	Discussion centers on patient-level changes
General suggestion	Suggestion that is not specific to the health system or the patient
**Provider, health team member**	**Discussion centers on the provider or health team member of discussion; role/specialty**
Radiology	Interactions with those in the radiology department
Pulmonology	Interactions with those in the pulmonology department
Primary care	Interactions with those involved in primary care
Oncology	Interactions with the oncology department
Surgery	Interactions with those involved in surgery
Navigator, coordinator	Interactions with navigators/coordinators
Nurse	Interactions with nurse
NP_PA	Interactions with nurse practitioner or physician assistant
Technician	Interactions with technicians
VISN	Interaction with VISN
Other provider or team member	interactions with other provider/health team members

**Practice_work experience**	**Designated columns**
Years of practice	How long have you been a __________(provider/staff/administrator) in _________ (specialty field)?
Other in last 5 years	Where else have you practiced/worked in the past 5 years?
Rural veteran percentage	percent of clinical practice live in rural area
Decision to implement personal involvement	Were you involved in or are you currently involved in the decision to implement a lung cancer screening program at your VA?
Involved in *decision* to implement	Were you involved in the decision to implement a lung cancer screening program at your VA? If yes, how were you involved?
Not involved in the *decision* to implement	Why were you not involved in the decision to implement a lung cancer screening program at your VA?
Status of implementation	Implemented/planning to implement
Implemented	lung cancer screening program is implemented
Planning to implement	Are you planning to implement a lung cancer screening program at your VA?
Other practice_work experience	Other practice or work experience not listed above
**World events**	**World events such as COVID-19 or political climate**
**Change over time**	**Discussion centers on change over time (e.g., Program evolve, VA quality of care)**
**Notable quotes**	**Quotes that stand out as having especially noteworthy content**
**Strategies and solutions for participation**	**Discussion centers around strategies used to educate the patient about the program**
Methods/information sources	Methods of education about the program
In person	Program education occur in person with provider/health team
Letter	Program education occur by way of postal mail
Monitor/television	Program education occur by way of waiting room television monitors
Posters	Program education occur by way of posters at the VA
Phone	Program education occur via phone call
My Healthy Vet	Program education occur via My Healthy Vet
Testimonials	Program education by way of testimonials
Other strategy or solution	Other strategy or solution
Shared decision making	Program enrollment involving shared decision making as strategy (intended to overcome barriers)
Setting expectations	Education includes setting realistic expectations
Resource coordination	Factors related to resource coordination (e.g., travel pay, screening site)
Rapport/trust/transparency/honesty	Individual mentions that creating a relationship between patient and provider helps create successful education and decision making
Appearance of presenter	Discusses demographic of person delivering information (if/how it matters) E.g., demographic, demeanor, etc.
Other strategy or solution/preference	Other strategy or solution/preference
Motivation to engage	Discussion centers on motivation to participate
Intrinsic	Discussion centers on intrinsic motivators (e.g., altruism)
Extrinsic	Discussion centers on extrinsic motivators (e.g., better outcomes)
